# The development and evaluation of a picture tongue assessment tool for tongue-tie in breastfed babies (TABBY)

**DOI:** 10.1186/s13006-019-0224-y

**Published:** 2019-07-16

**Authors:** Jenny Ingram, Marion Copeland, Debbie Johnson, Alan Emond

**Affiliations:** 1 Centre for Academic Child Health, Bristol Medical School, University of Bristol, 1-5 Whiteladies Road, Bristol, BS8 1NU UK; 2 Maternity Department, Southmead Hospital, North Bristol NHS Trust, Bristol, BS10 5NB UK

**Keywords:** Tongue-tie, Frenotomy, Breastfeeding

## Abstract

**Background:**

The presence of a tongue-tie (ankyloglossia) in an infant may lead to breastfeeding difficulties, but debate continues about which babies should be treated with frenotomy. The Bristol Tongue Assessment Tool (BTAT), a clear and simple evaluation of the severity of tongue-tie, is being used worldwide and translated into different languages. We aimed to produce a simple picture version of the BTAT to aid and enhance consistent assessment of infants with tongue-tie.

**Methods:**

The Tongue-tie and Breastfed Babies (TABBY) assessment tool was developed from the BTAT by a graphic designer, with iterative discussion with four practicing NHS midwives. The TABBY tool consists of 12 images demonstrating appearance of the infant tongue, its attachment to the gum and the limits of tongue mobility. The TABBY tool is scored from 0 to a maximum of 8.

Two initial audits of the TABBY were undertaken at a large maternity unit in a secondary care NHS Trust, in Bristol UK from 2017 to 2019. TABBY was evaluated by five midwives on 262 babies with tongue-ties and experiencing breastfeeding difficulties who were referred for assessment to a tongue-tie assessment clinic using both BTAT and TABBY. Each pair of scores was recorded by one midwife at a time. A further training audit with 37 babies involved different assessors using BTAT and TABBY on each baby.

**Results:**

All midwives found the TABBY easy to use, and both audits showed 97.7% agreement between the scores. We suggest that a score of 8 indicates normal tongue function; 6 or 7 is considered as borderline and 5 or below suggests an impairment of tongue function. Selection of infants for frenotomy required an additional breastfeeding assessment, but all infants with a score of 4 or less in the audits had a frenotomy, following parental consent.

**Conclusions:**

The TABBY Assessment Tool is a simple addition to the assessment of tongue-tie in infants and can provide an objective score of tongue-tie severity. Together with a structured breastfeeding assessment it can inform selection of infants for frenotomy. It can be used by clinical staff following a short training and will facilitate translation into other languages.

## Background

The presence of a tongue-tie (ankyloglossia) in an infant may lead to breastfeeding difficulties, but debate continues about which babies should be treated with frenotomy [1, 2]. Breast and bottle-feeding difficulties have been reported in 25–44% of infants with tongue-tie and these include poor attachment, inability to breastfeed continuously, unsettled infants with poor weight gain and maternal nipple trauma and pain [3].

Dividing the tongue-tie, frenotomy, is a simple procedure in the young infant that can be performed without anaesthetic and with few complications [4]. Those performing frenotomy should have received appropriate training and it is essential that both clinical and on-going lactation support for women are provided. Infants with tongue-tie who are having difficulties breastfeeding, despite support with breastfeeding, could possibly benefit from division of the frenulum to facilitate the initiation and maintenance of exclusive breastfeeding.

A systematic review of studies reporting the effects of frenotomy on breastfeeding concluded that tongue-tie division improves many aspects of breastfeeding for most newborn infants and their mothers [5]. A further review reported that frenotomy may be associated with mother-reported improvements in breastfeeding and nipple pain, but the strength of the evidence for sustained improvement in breastfeeding rates is low [3].

To identify infants who would benefit from frenotomy, two assessments are required: a review of breastfeeding efficacy by a trained practitioner using a structured assessment tool and an objective assessment of the severity of the tongue-tie. In 2012 we produced a simple assessment tool with good transferability to provide consistent assessment of tongue appearance and function for infants with tongue-tie identified in the early weeks [6]. The Bristol Tongue Assessment Tool (BTAT) provides an objective, clear and simple evaluation of the severity of the tongue-tie. Following publication in 2015, there has been considerable interest in using the BTAT from health professionals around the world, including New Zealand, USA, Finland and Brazil. In Canterbury, New Zealand, use of the BTAT in infants with breastfeeding difficulties was associated with a reduction in frenotomy rates from 11.3 to 3.5% over a two-year period [7]. The tool is being used by a variety of health professionals and has been translated into different languages, including Finnish and Portuguese; this has led us to develop a picture version with short descriptive headings to make is easier to translate and use in other countries. We are also frequently asked for recommendations about how to use the score produced to guide selection for frenotomy and so our suggestions have been included in the paper.

This paper describes the development and initial evaluation of the new pictorial Tongue-tie and Breastfed Baby (TABBY) assessment tool.

## Methods

### Development and evaluation of TABBY compared to BTAT

**Fig. 1:**
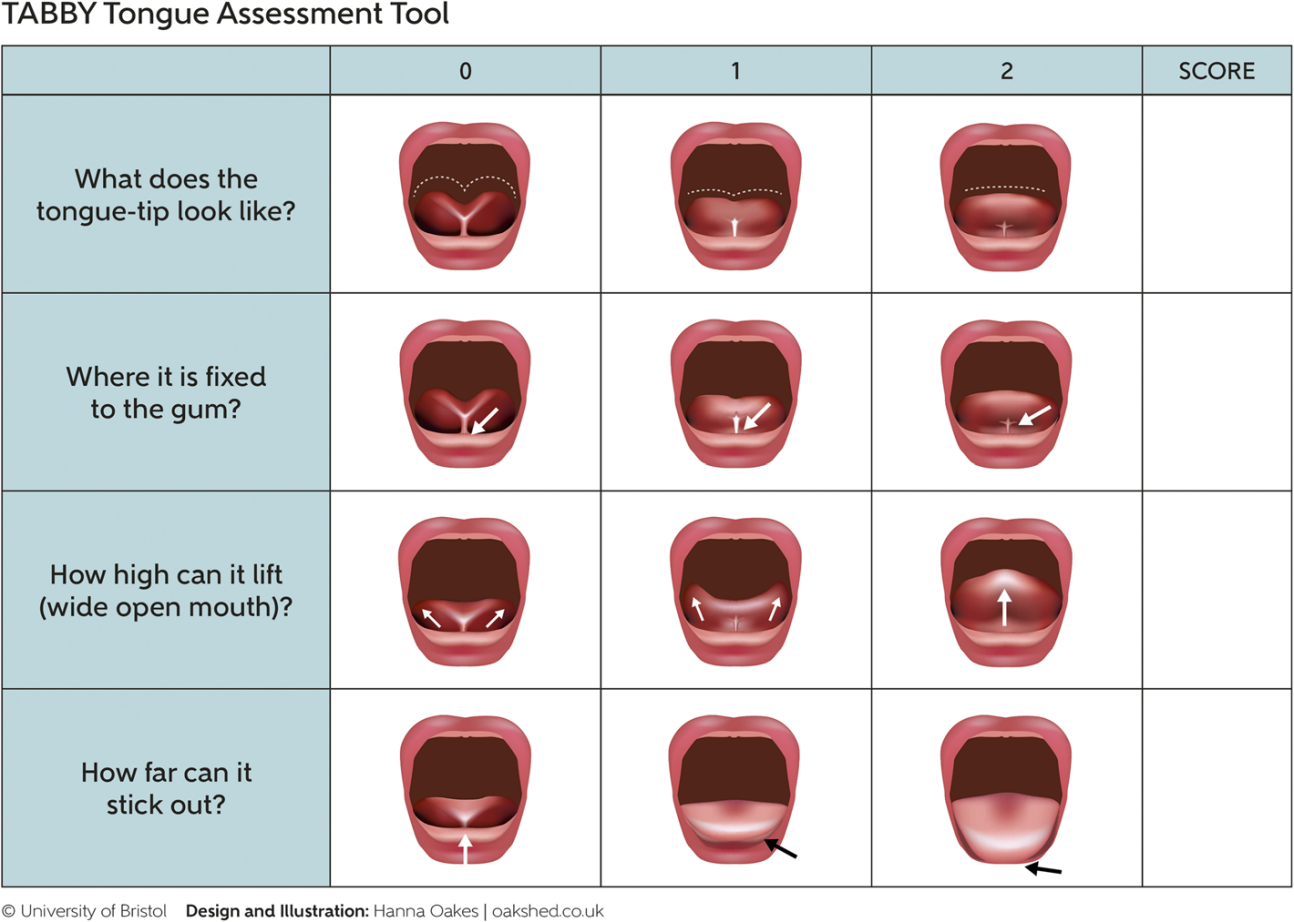
TABBY assessment tool

A graphic designer was commissioned to illustrate the 12 boxes on the BTAT under the four descriptions of ‘What does the tongue-tip look like?’, ‘Where is it fixed to the gum?’, ‘How high can it lift (wide open mouth)?’, and ‘How far can it stick out?’ to produce the TABBY tool (Fig. 1). An iterative discussion with four practicing NHS midwives refined the illustrations over a period of 6 weeks and the final version was then tested at a busy tongue-tie assessment clinic in one secondary care NHS Trust. Table 1 provides guidance about using the TABBY tool.

**Table 1:** Guidance on the use of the TABBY (Tongue-tie and Breastfed Babies) assessment tool

TABBY category	Guidance on use of TABBY
What does the tongue-tip look like	This is usually the most obvious and most likely to be noted by parents. A notch in the tip of the tongue may only be noticed when the baby lifts the tongue.
Where it is fixed to the gum?	With some training and experience this can be assessed visually. If it is difficult to see, then the assessor can [with parental consent] gently use their index finger to feel where the frenulum is attached.
How high can it lift (wide open mouth)?	This can be the most difficult to teach. The assessor needs awareness of normal tongue lift in infants.
	The tongue may curl back when restricted and so appear to lift. The lift is most easily viewed if the infant is awake and crying. If the baby is not awake, then the assessor can digitally lift the tongue to assess.
How far can it stick out?	This is not always easy to assess in newborn infants. It can be helpful to ask parents what they have noticed, and the pictures can be helpful in discussing this. The easiest way to assess protrusion is to watch the baby as they latch to the breast; are they able to bring the tongue out to latch?

### Evaluation audits

Evaluation 1: Five midwives assessed 262 babies with tongue-ties from August 2017 to March 2018 and compared their scores using both BTAT and TABBY. Most of the comparisons were conducted by the senior infant feeding midwife who runs the tongue-tie assessment clinic with four other midwives each conducting 10 to 20 assessments. Each pair of scores was recorded by one midwife at a time. Scores were compared using weighted kappas.

Evaluation 2: This was conducted with 37 babies at the frenotomy clinic to assess how well the tool could be used in training. Two experienced midwives used the BTAT to assess tongue function of a baby whilst a less experienced midwife or health care assistant (not familiar with BTAT) used the TABBY to assess each baby during December 2018 and January 2019.

A correlation coefficient between scores in the same baby was estimated using a mixed-effects model with tool and assessor as fixed effects, and baby as a random effect.

## Results

Evaluation 1: All five midwives involved in the first audit found the TABBY easy to use, and there was 97.7% agreement (overall weighted kappa 0.923) between the scores. Table 2 shows the correlation between the BTAT and TABBY scores.

**Table 2:** Comparison of BTAT (Bristol Tongue Assessment Tool) and TABBY (Tongue-tie and Breastfed Babies assessment tool) scores

	BTAT									
TABBY	0	1	2	3	4	5	6	7	8	Total
0	**22**	1								23
1		**8**	3							11
2		1	**13**	4						18
3			1	**11**	2					14
4				5	**45**	3				53
5				1	3	**35**	7			46
6						7	**36**	3		46
7							6	**34**	1	41
8									**10**	10
Total	22	10	17	21	50	45	49	37	11	262

Evaluation 2: For the training audit, correlation between the two tools, taking into account the use of four different assessors, was 0.978 (95% CI 0.958 to 0.988), which is consistent with the high level of agreement shown in Evaluation 1.

**Fig. 2:**
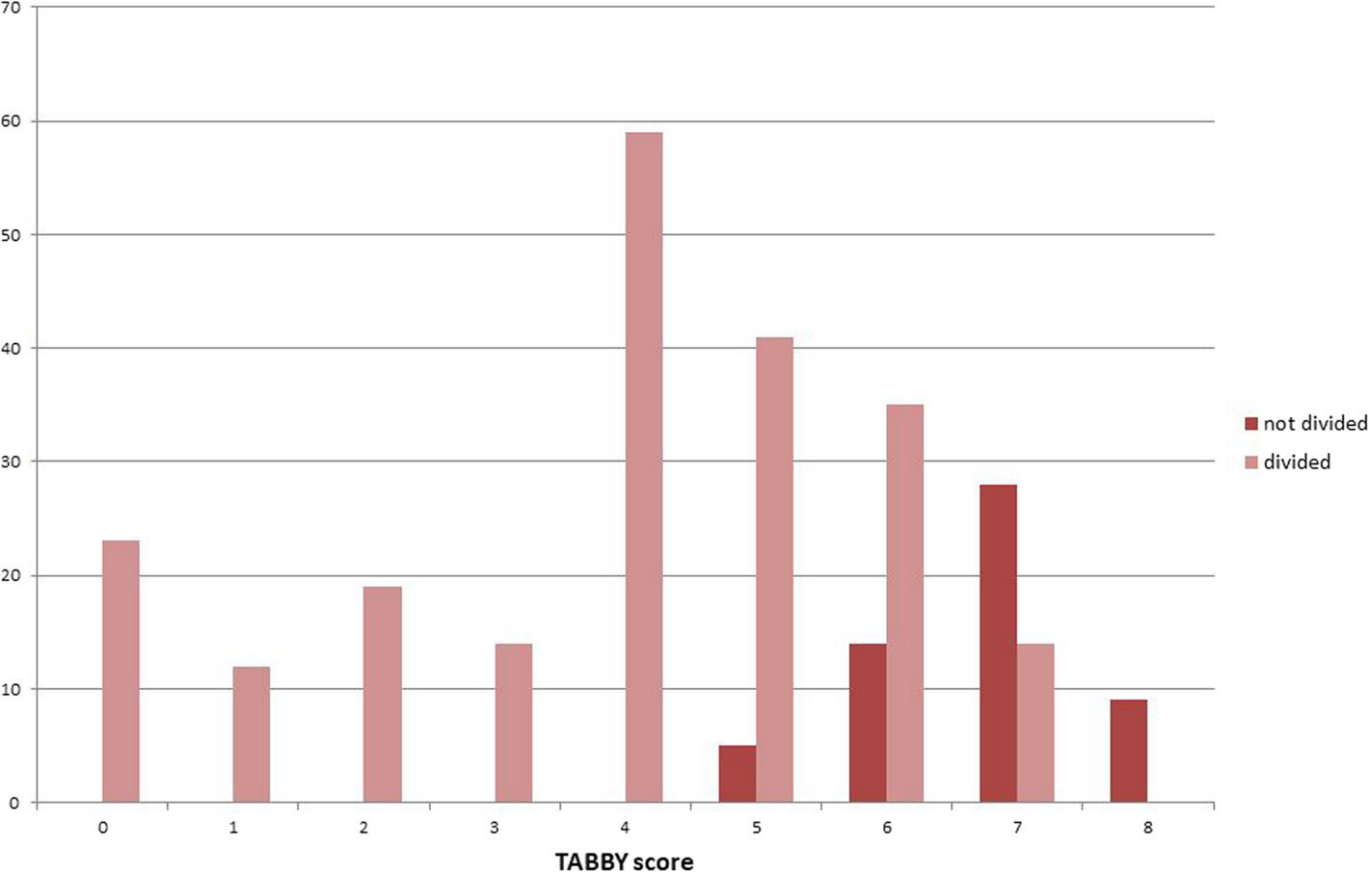
BTAT/TABBY score range and whether frenotomy was conducted

Figure 2 shows the association between BTAT/TABBY score and whether a tongue-tie was divided or not. It indicates that all scores of 4 and below were divided and most of those scoring 5; two-thirds of score 6 and one-third of score 7 were divided and none of those scored as 8.

### Scoring of BTAT or TABBY tool

Iterative discussions between staff in two NHS Trusts using the BTAT since 2016 have helped to refine the cut off recommendations for frenotomy. The collective experience of these midwives, infant feeding specialists, paediatricians, ENT surgeons and breastfeeding experts using the BTAT and more recently the TABBY tool to assess over 2000 babies suggests that a score of:

- 8 indicates normal tongue function;
- 6 or 7 are considered as borderline: suggest a ‘wait and see’ approach with support for breastfeeding positioning & attachment;
- 5 or below suggests that there is impairment of tongue function: this may or may not be having an effect on breastfeeding.

### Selection of infants for frenotomy

Assessment of tongue function is only one part of the feeding assessment and so the decision to divide a tongue-tie should be based on:

- assessment of breastfeeding: is there a feeding problem?
- assessment of tongue structure and function using BTAT/TABBY: is the tongue movement restricted?
- clinical judgement: is the feeding problem caused by the tongue-tie; considering maternal anatomy?
- discussion with parents: not all parents want the tongue-tie to be divided.

For the feeding assessment it is essential to observe a breastfeed: we use the UNICEF BFI assessment tool [8] and the Bristol Breastfeeding Assessment Tool [9]. This feeding assessment can be done by any *trained* health professional, but the decision to divide or not should be made by the health professional who is dividing the tongue-tie with the informed consent of the parents. If midwives or breastfeeding specialists are not trained to divide tongue-tie then it is best to use the assessment process above and discuss with parents, without making a judgement about whether the tongue-tie should be divided or not.

## Discussion

We have produced a pictorial version of an established clinical tool to assess the severity of ankyloglossia and undertaken two initial audits in a single centre. The picture version of the tongue assessment tool works well and is a useful addition to the assessment of tongue-tie in infants. It is quick and easy to use, can be used as a training tool and is straightforward to translate into other languages. The TABBY only assesses tongue structure and function and not the impact on feeding. When used it should be combined with an evaluation of breastfeeding using a structured assessment tool, and a discussion with the mother about the comfort and perceived efficacy of breastfeeding.

The strengths of the TABBY are that it is easy to use and provides a visual aid to help the assessor be clear about crucial features of a tongue tie: the appearance of the tongue tip, the insertion of the frenulum and the mobility of the tongue (lift and protrusion). Using a small sample of midwives, the TABBY was scored in a consistent way, and when combined with a breastfeeding assessment, helped to identify infants for frenotomy. Midwives also reported that the picture version was helpful when describing their baby’s tongue function to parents.

The main limitation of the TABBY is the same as for the BTAT from which it was derived- it cannot be used alone to select infants for frenotomy as it does not include any assessment of feeding, for which a separate evaluation is needed using a structured tool, consideration of maternal anatomy, and a sensitive interview with the breastfeeding mother. In this study, we were not able to determine what effect the use of the TABBY rather than the BTAT would have on frenotomy rates- further audits will be needed in the future with larger numbers in different clinical settings.

The TABBY is intended to be used as part of the *initial* assessment of a perceived tongue-tie in a busy delivery suite, postnatal ward or community clinic, and has advantages over existing assessment tools in that it can be used by midwives, health care assistants and medical staff after a short training, it is quick to use and simple to score. The Coryllos classification is a simple 4-point scale based on the attachment site of the frenulum to the tongue and alveolar ridge but does not assess tongue function [10]. The more comprehensive Assessment Tool for Lingual Frenulum Function (ATLFF) [11] produces appearance and function scores and is suitable for use by lactation specialists or in private practice. The Lingual Frenulum Protocol with Scores for Infants [12] is a two-part assessment designed for use by speech and language therapists, which takes longer to complete because it has questions about breastfeeding efficacy and pain as well as an evaluation of tongue appearance and function.

We hope that the findings of this initial evaluation of the TABBY will be reproducible in other tongue-tie assessment settings, and that the new picture tool will be useful in research studies. Our recommendations for the use of TABBY are built on experience with the BTAT and supported by Dixon et al. in New Zealand [7] who have integrated the BTAT into their clinical care pathway for assessing breastfeeding problems and shown that this has led to more appropriate referrals for frenotomy being made. We plan to evaluate the use of the TABBY tool in other languages and in different settings.

## Conclusions

The TABBY Assessment Tool is a clear and simple addition to the assessment of tongue-tie in infants and can provide an objective score of the severity of a tongue-tie. Together with a structured breastfeeding assessment it can inform selection of infants for frenotomy. It can be used by clinical staff following a short training and will facilitate translation into other languages.

## Data Availability

The datasets used and/or analysed during the current study are available from the corresponding author on reasonable request.
